# HPV infection in women with and without cervical cancer in Conakry, Guinea

**DOI:** 10.1038/sj.bjc.6605140

**Published:** 2009-06-16

**Authors:** N Keita, G M Clifford, M Koulibaly, K Douno, I Kabba, M Haba, B S Sylla, F J van Kemenade, P J F Snijders, C J L M Meijer, S Franceschi

**Affiliations:** 1 Department of Obstetrics and Gynaecology, Centre Hospitalier Universitaire de Donka, B.P. 921, Conakry, Guinea; 2 International Agency for Research on Cancer, 150 cours Albert Thomas, 69372 Lyon cedex 08, France; 3 Department of Pathology, Centre Hospitalier Universitaire de Donka, B.P. 921, Conakry, Guinea; 4 Department of Pathology, Vrije University Medical Center, Postbus 7057, 1007 MB Amsterdam, The Netherlands

**Keywords:** cervical cancer, human papillomavirus, prevalence, Guinea, Africa

## Abstract

**Background:**

Cervical cancer incidence in western Africa is among the highest in the world.

**Methods:**

To investigate human papillomavirus (HPV) infection in Guinea, we obtained cervical specimens from 831 women aged 18–64 years from the general population of the capital Conakry and from 77 locally diagnosed invasive cervical cancers (ICC). Human papillomavirus was detected using a GP5+/6+ PCR-based assay.

**Results:**

Among the general population, the prevalence of cervical abnormalities was 2.6% by visual inspection and 9.5% by liquid-based cytology. Fourteen of 15 high-grade squamous intraepithelial lesions were visual inspection-negative. Human papillomavirus prevalence was 50.8% (32.1% for high-risk types) and relatively constant across all age groups. Being single or reporting ⩾3 sexual partners was significantly associated with HPV positivity. HPV16 was the most common type, both among the general population (7.3%) and, notably in ICC (48.6%). HPV45 (18.6%) and HPV18 (14.3%), the next most common types in ICC, were also more common in ICC than in HPV-positive women with normal cytology from the general population.

**Conclusion:**

The heavy burden of HPV infection and severe cervical lesions in Guinean women calls for new effective interventions. Sixty-three per cent of cervical cancers are theoretically preventable by HPV16/18 vaccines in Guinea; perhaps more if some cross-protection exists with HPV45.

The Republic of Guinea in western Africa has a population of approximately 9.4 million, of which 2 million live in the capital of Conakry. The country shows high birth rates (5.8 children per woman) and communicable diseases, notably malaria, which predominate as causes of death. Estimates of cervical cancer incidence and mortality rates in western Africa are among the highest in the world (Ferlay *et al*, 2004), and in Conakry, cervical cancer is by far the most common malignancy in women (Koulibaly *et al*, 1997).

Establishing the viral aetiology of cervical cancer has raised the hopes for primary and secondary prevention through human papillomavirus (HPV) vaccination (Kahn and Burk, 2007) and HPV DNA test-based screening (Cuzick *et al*, 2006), respectively. The rationale and planning of such measures require population-based epidemiological data on overall and type-specific HPV prevalence in women with and without cancer. To this end, the International Agency for Research on Cancer (IARC) has carried out surveys in representative samples of women worldwide (Clifford *et al*, 2005).

We report on HPV prevalence survey among a representative sample of the general female population in Conakry, Guinea, as well as in a corresponding sample of Guinean women with invasive cervical cancer (ICC).

## Materials and methods

Study methods were similar to those used for earlier IARC HPV Prevalence Surveys (Clifford *et al*, 2005). In the study area of Gbessia Port, a densely populated district of Conakry, we aimed to enrol approximately 100 women from the general population in each 5-year age group between 15 and 19 and 60 and 64 years. All mentally and physically competent women, regardless of their marital and sexual activity status, were eligible. Women were enumerated at their homes by local community workers and invited to the Gbessia Port Health Centre (Centre de Santé de Gbessia Port) between April and December 2006 to participate in the study.

Of the 1725 invited women, 440 (25.5%) did not accept the invitation, mainly because they apparently did not understand the need for gynaecological examination in the absence of symptoms. Acceptance was similar in all the age groups, but owing to the age structure of the population, relatively few women over 44 years of age could be invited (life expectancy of women in Guinea was estimated in 2004 to be 55 years [www.who.int]). In addition, among the 462 women below age 25 years who accepted the invitation to the study clinic, 213 (of whom 148 were self-declared virgins) were unwilling to undergo a pelvic examination, and opted to provide only a blood sample. Hence, no cervical cell specimen was collected for these 213 women; an additional 73 women aged 25 years or older did not undergo a pelvic examination mainly because of heavy menstrual bleeding.

A structured questionnaire was administered by one of the five nurses in the local dialect covering socio-demographic characteristics, reproductive and menstrual factors, sexual habits, including those of the participants and their husbands’ and lifetime use of contraceptive methods.

A total of 999 women underwent a pelvic examination by one of the three midwives. Three women presented with an advanced cervical cancer that did not allow for collection of an adequate cervical cell sample. Among the remaining 996, a sample of exfoliated cervical cells for liquid-based cytology and HPV testing was collected. A cytobrush, after insertion into the endocervical canal, was rotated gently at 180° to collect cells, was then placed with its cellular material in a vial containing PreservCyt media (Cytyc-Hologic, Marlborough, MA, USA). The cervix was then inspected with acetic acid (VIA) and visual inspection with Lugol's iodine (VILI), with results reported according to the IARC criteria (Sankaranarayanan and Wesley, 2003).

Slides for liquid-based cytology were prepared using a Thin Prep 3000 processor (Cytyc-Hologic), stained according to manufacturer's instructions and read at the Department of Pathology at the Vrije University Medical Center, Amsterdam, the Netherlands. Cytological diagnosis was formulated according to CISOE-A standards and was translated into the Bethesda 2001 terminology system (Bulk *et al*, 2004). Confirmatory biopsies, when available, were read (MK) in the Centre National d’Anatomie Pathologique, Centre Hospitalier Universitaire (CHU) de Donka.

Women presenting with ICC at the gynaecological clinic of the CHU de Donka, Conakry, were identified between January 2006 and March 2007. The collection of a tumour biopsy for study purposes was possible for all 99 women diagnosed with ICC; most were classified as stage II (51 women) or III (44 women). Biopsies were fixed with buffered formalin, embedded in paraffin (MH), then read (MK) in the Centre National d’Anatomie Pathologique, CHU de Donka. HIV testing was available for 41 women with ICC, of which eight (19.5%) were HIV-positive; among 19 women with ICC below age 45 years, six were HIV-positive.

All participants, whether from the HPV prevalence survey or those with ICC, signed informed consent forms according to the recommendations of the IARC and the CHU de Donka ethical review committees, both of which approved the study.

HPV testing was performed on exfoliated cervical cells and ICC biopsies in the Department of Pathology at the Vrije University Medical Center. DNA was extracted from the PreservCyt sample using magnetic beads (Macheri-Nagel, Düren, Germany) on a robotic system (Hamilton, Germany), according to the manufacturer's instructions. Invasive cervical cancer biopsies were sectioned using a ‘sandwich’ approach, whereby inner tumour sections were destined for HPV testing and outer sections for histological confirmation of tumour tissue. One or more five lM sections representing approximately 1 cm^2^ of tissue were pre-digested with Proteinase K after which DNA was extracted using magnetic beads (Macheri-Nagel, Germany).

Beta-globin PCR analysis was performed first to confirm the presence of human DNA in all specimens (Roda Husman *et al*, 1995). The overall presence of HPV DNA was determined by performing a general primer GP5+/6+-mediated PCR, which permits the detection of a broad spectrum of genital HPV types at the subpicogram level (Jacobs *et al*, 2000). Human papillomavirus positivity was assessed by hybridisation of PCR products in an enzyme immunoassay using two HPV oligoprobe cocktails that, together, detect the following 44 HPV types: HPV6, 11, 16, 18, 26, 30, 31, 32, 33, 34, 35, 39, 40, 42, 43, 44, 45, 51, 52, 53, 54, 55, 56, 57, 58, 59, 61, 64, 66, 67, 68, 69, 70, 71 (equivalent to CP8061), 72, 73, 81 (equivalent to CP8304), 82 (IS39 and MM4 subtypes), 83 (equivalent to MM7), 84 (equivalent to MM8), cand85, 86, cand89 (equivalent to CP6108) and JC9710. Subsequent HPV typing was performed by reverse line blot hybridisation of PCR products, as described earlier (van den Brule *et al*, 2002). In this typing format, all aforementioned HPV types were individually genotyped, except for the uncommon HPV types 32, 83, 84, 85, 86 and JC9710. Oligoprobes representing the latter types were loaded as a pool on the reverse line blots and consequently were genotyped as a pool. Human papillomavirus types considered high risk for this analysis that comprised HPV16, 18, 31, 33, 35, 39, 45, 51, 52, 56, 58, 59, 68, 73 and 82 (Muñoz *et al*, 2003); all other HPV types were considered low risk.

### Statistical analysis

Odds ratios (ORs) for HPV positivity and corresponding 95% confidence intervals (CIs) were calculated by means of unconditional logistic regression equations, adjusted for age group (15–24, 25–34, 35–44, 45–54, 55–64 years), marital status (never- or ever-married) and lifetime number of sexual partners (1, 2 and ⩾3), as appropriate. The statistical significance of trends for ORs was assessed by considering the categorical variable as a continuous variable in the logistic model. Prevalence ratios and corresponding 95% CIs were used to compare the relative frequency of the most common HPV types in HPV-positive women with ICC with that among cytologically normal HPV-positive women from the HPV prevalence survey.

## Results

Of 996 women who provided cervical cell samples, 84 had inadequate HPV DNA results (77 *β*-globin-negative, seven missing), and an additional 81 had inadequate cytology, leaving 831 women with valid results for both HPV and cytology. Among them, 79 (9.5%) had abnormal cytological findings, including 48 (5.8%) atypical squamous cells of undetermined significance (ASCUS), 16 (1.9%) low-grade squamous intraepithelial lesions (LSIL) and 15 (1.8%) HSIL.

Overall HPV prevalence was 50.8% (78.5% and 47.9% among women with and without cervical abnormalities, respectively, Table 1). The corresponding age-standardised prevalence to the world population was 51.5% (95% CI: 48.0–55.0). In total, 275 (33.1%) women had single-type and 147 (17.7%) had multiple-type infections. Prevalence of high-risk and low-risk types (32.1, 30.5%, respectively) was similar. The commonest high-risk types with normal cytology were HPV16 (6.7%), 45 (4.7%), 52 (4.0%), and 18, 35 and 58 (3.2% each). HPV66, 42 and 81 were the most commonly detected low-risk types. HPV16 was also the commonest type (13.9%) among women with cervical abnormalities, of whom 59.5% had high-risk types. The prevalence of high-risk HPV types in women with HSIL or worse was 73.3% (data not shown).

Twenty-one women had abnormalities suspected at VIA/VILI. The correlation between the results of VIA/VILI and those of liquid-based cytology and HPV testing was poor (Table 2). VIA/VILI identified one HSIL (that was shown to be ICC by histology), but missed 14 of 15 HSIL, 15 of 16 LSIL and all 48 ASCUS. The proportion of suspected abnormalities at VIA/VILI did not differ significantly between high-risk HPV-positive (7/264; 2.7%) and high-risk HPV-negative (14/558; 2.5%) women (*χ*^2^_1_=0.0146, *P*=0.904).

Of the 21 women with suspected abnormalities at VIA/VILI, 17 accepted referral to colposcopy and 10 had colposcopy-directed biopsies, none of which showed lesions except the aforementioned ICC. Repeated attempts were also made after cytological findings became available (i.e., approximately one year and a half after the initial visit) to recall the 14 women who had no abnormality detected at VIA/VILI but HSIL found at liquid-based cytology. Three women, however, had moved far from Conakry, one was not found at her address, one had died of a disease other than cervical cancer and one was in the third trimester of pregnancy. Of the eight biopsies taken, one was inadequate, three showed cervical intraepithelial neoplasia 1 and the other four showed no cervical lesion.

**Figure 1:**
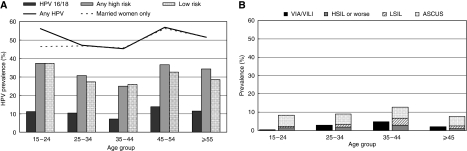
Age-specific prevalence of (**A**) HPV DNA by HPV type(s) overall (831 women) and among married women only (674 women) (**B**) cervical abnormalities at VIA/VILI and liquid-based cytology. Conakry, Guinea, 2006–2008. ASCUS, atypical squamous cells of undetermined significance; HPV, human papillomavirus; HSIL, high-grade squamous intraepithelial lesion; LSIL, low-grade squamous intraepithelial lesion; VIA, visual inspection with acetic acid; VILI, visual inspection with Lugol's iodine.

Figure 1A and B show, respectively, the prevalence of HPV (any HPV type, HPV16 and/or 18 and any high-risk types and low-risk types, separately) and of VIA/VILI and cytological abnormalities by age group. Human papillomavirus prevalence was 56.1% among women younger than 25 years, and dropped to 45.3% in women aged 35–44 years, before increasing again up to 55% in women aged 45 years or older. Age-specific patterns were similar for the prevalence of HPV16 and/or 18 and any high-risk type. When 157 women who had never been married (75.8% of whom were <25 years) were excluded, HPV prevalence below age 25 years decreased to 46.3% (Figure 1A). VIA/VILI and cytological abnormalities increased up to age 35–44 years and then declined.

Table 3 shows the relationship between HPV positivity and various characteristics of participants according to two different models. In the age-adjusted model, significant differences in HPV positivity were observed by age group (OR for 35–44 *vs* <25 years=0.65; 95% CI: 0.44–0.95), marital status (OR in single *vs* married women=1.60; 95% CI: 1.04–2.45) and number of lifetime sexual partners (OR for ⩾3 *vs* 1 partner=1.71; 95% CI: 1.03–2.84). These three associations were slightly attenuated in the model additionally adjusted, as appropriate, for marital status and sexual partners.

Education, number of births, age at first sexual intercourse, husbands’ polygamy or extra-marital sexual relationships and lifetime use of hormonal contraceptives were unrelated to HPV positivity in either adjustment model (Table 3) as were smoking habits, condom use (reported by only 16 women) and history of spontaneous or induced abortions (data not shown). Only six (0.7%) participants reported having had previous cervical cancer screening.

Beta-globin-negative ICC biopsies (22) were excluded; among the remaining 77 ICC biopsies with valid HPV results, 8 were adeno- and the rest were squamous-cell carcinoma. The median age of women with ICC was 45 years (range: 23–80 years). Seven ICC biopsies, including one adenocarcinoma, were HPV-negative. Type-specific HPV prevalence in 70 HPV-positive ICC and 360 cytologically normal HPV-positive women from the HPV prevalence survey are compared in Table 4. HPV16 was found in 34 (48.6; 95% CI: 36.4–60.8%) HPV-positive ICC biopsies. The next commonest types were HPV45 (18.6; 95% CI: 10.3–29.7%) and 18 (14.3; 95% CI: 7.1–24.7%). Prevalence ratios in women with ICC *vs* cytologically normal women were 3.5 (95% CI: 2.2–5.5) for HPV16, 2.1 (95% CI: 0.9–4.6) for HPV18; 1.9 (95% CI: 0.9–3.7) for HPV45 and 0.8 (95% CI: 0.5–1.2) for high-risk types other than 16 or 18. Multiple-type infections were much less common in HPV-positive women with ICC than in HPV-positive women with normal cytology (Table 4). Among the five ICC biopsies deriving from HIV-infected women with a valid HPV result, HPV16, 45, 18 were found in two, two and one woman, respectively.

## Discussion

Our large study, the first on HPV infection in Guinea, showed a very high (50.8%) prevalence of HPV, and nearly no history of cervical cancer screening. Approximately one-third of women in our survey were infected with high-risk HPV types and four prevalent ICC were discovered.

The age-standardised HPV prevalence we found in Conakry (51.5%) was considerably higher than that observed in earlier studies performed using the same HPV testing protocol in areas at high risk for cervical cancer such as South America (12–18%) and India (17%) or even another western African population in Nigeria (27%) (Franceschi *et al*, 2006). It is also of the highest HPV prevalence reported in any study in western Africa (Xi *et al*, 2003; Wall *et al*, 2005; Lack *et al*, 2005) or other parts of sub-Saharan Africa (de Sanjosé *et al*, 2007; Castellsagué *et al*, 2008), with the exception of those that included only, or a majority of, HIV-positive women (Sahasrabuddhe *et al*, 2007). Differences in age and sensitivity of PCR testing methods, however, limit the direct comparability with studies other than the IARC HPV Prevalence Surveys.

Nearly half of study women reported having two sexual partners or more in their lifetime, and over two-thirds reported husband's polygamy or extra-marital relationships. These indicators of high-risk sexual behaviour can help to explain the elevated HPV prevalence in this study. Conversely, on account of the very high frequency of HPV infection that emerged in our study, it is not surprising that HPV-negative and -positive women did not differ by various characteristics, including education level, except for a moderately increased infection risk in single women and women who reported three sexual partners or more in their lifetime.

An influence of undetected HIV infection on high HPV prevalence in our study women cannot be ruled out, as the protocol of the IARC HPV Prevalence Surveys does not include HIV testing. In 2004, a survey of pregnant women estimated the prevalence of HIV to be 4.2% nationally, and 6% in Gbessia Port, Conakry; this seemed to be fairly uniform across age groups, implying a well-established epidemic, but was somewhat higher among unmarried women (9.2%) (Ministère de la Santé Publique, 2005). HIV infection among tested women with ICC (20%) was high considering the limited life expectancy of people with HIV/AIDS during the study period (Franceschi and Jaffe, 2007).

One of the main aims of the IARC HPV Prevalence Surveys concerns the variations of age-specific and type-specific HPV prevalence by geographical region. The age-specific curve of HPV prevalence in Guinea resembled the age curves reported earlier in Nigeria (Thomas *et al*, 2004), India (Franceschi *et al*, 2005) and China (Dai *et al*, 2006; Li *et al*, 2006; Wu *et al*, 2007), but differed from the steep decrease in prevalence seen by age in high-resource countries (Franceschi *et al*, 2006; De Vuyst *et al*, 2009). A modest peak in women below age 25 years in Guinea was accounted for by the participation of unmarried women in this age group (Franceschi *et al*, 2006). By contrast, in IARC HPV Prevalence Surveys in India and China it was considered socially unacceptable to invite unmarried women (Franceschi *et al*, 2005; Dai *et al*, 2006; Li *et al*, 2006; Wu *et al*, 2007). In some earlier studies from sub-Saharan Africa, HPV prevalence also remained high (Kuhn *et al*, 2000; Thomas *et al*, 2004; Wall *et al*, 2005) or even increased (Xi *et al*, 2003) in middle and old age, whereas peaks in young women were reported by other investigators (Serwadda *et al*, 1999; Womack *et al*, 2000; Castellsagué *et al*, 2001; De Vuyst *et al*, 2003; Baay *et al*, 2004).

With respect to the relative importance of different HPV types, HPV16 was the commonest type overall, that is, 13.9%, 17.7% and 48.6%, respectively, in cytologically normal and abnormal women and in women with ICC (Clifford *et al*, 2005; Smith *et al*, 2007). Noticeably, however, the high proportion of HPV45 (18.6; 95% CI: 10.3–29.7%) is consistent with a meta-analysis of HPV type distribution in ICC from western Africa (Smith *et al*, 2007). Elsewhere, HPV45, a high-risk type that, similar to HPV18, belongs to the *α*-7 species, is usually less frequently detected than HPV18 in cytologically normal women (de Sanjosé *et al*, 2007) and women with ICC (Smith *et al*, 2007).

On account of the lack of experienced cytologists and HPV testing in Conakry, we relied on VIA/VILI for the immediate clinical management of study women from the HPV prevalence survey. Despite the substantial experience of study midwives in visual inspection (Sankaranarayanan *et al*, 2004), the majority (77/79) of cytological abnormalities later showed by liquid-based cytology were VIA/VILI-negative (including 14/15 HSIL), and 61.9% of abnormalities suspected at VIA/VILI were found in cytologically normal women who were negative for high-risk HPV types. None of the 10 biopsies taken among VIA/VILI-positive women, with the exception of one ICC case, showed cervical intraepithelial neoplasia of any grade.

Major strengths of our study include the large number of women, high participation, reliance on high-quality HPV testing and liquid-based cytology, and presence of a concurrent series of women with ICC drawn from the same study area. Limitations include the lack of information on HIV status and the relatively high proportion of *β*-globin-negative samples in the HPV prevalence survey, and of HPV-negative findings in paraffin-embedded ICC biopsies. Most important, the lack of prompt histological confirmation of abnormalities and of random biopsies did not allow us to accurately estimate sensitivity and specificity of different methods. A prolonged curfew in 2007 delayed not only the shipment of cervical cell samples to Europe and, hence, the availability of cytological and HPV findings, but also the recall of women. Substantial population mobility and scarcity of pathology staff in Conakry also hampered the appropriate work up of screening positive women.

The very heavy burden of HPV infection and severe cervical lesions in Guinea calls for new effective interventions. The quality of VIA/VILI should be greatly improved, or other types of screening used (e.g., rapid HPV test, Qiao *et al*, 2008), whereas screen-and-treat approaches should be encouraged for recalling women. With respect to the potential benefit of vaccination, the fraction of ICC preventable in Guinea (62.9; 95% CI: 50.5–74.1) by a vaccine including HPV16/18, is compatible with the worldwide estimate (Smith *et al*, 2007), but would be substantially improved if some cross-protection between HPV16/18 and HPV45 was confirmed to exist (Barr and Sings, 2008; Jenkins, 2008).

## Figures and Tables

**Table 1:** Prevalence of HPV types by cytological findings and overall among 831 women (Conakry, Guinea, 2006–2008)

	**Normal cytology (*N*=752)**			**Abnormal cytology (*N*=79)**			**Total (*N*=831)**		
**HPV type**	**Single**	**Multiple**	**Total (%)**	**Single**	**Multiple**	**Total (%)**	**Single**	**Multiple**	**Total (%)**
HPV−	—	—	392 (52.1)	—	—	17 (21.5)^a^	—	—	409 (49.2)
HPV+	241	119	360 (47.9)	34	28	62 (78.5)	275	147	422 (50.8)
High-risk HPV+	118	102	220 (29.3)	23	24	47 (59.5)	141	126	267 (32.1)
Low-risk HPV+	123	96	219 (29.1)	11	23	34 (43.0)	134	119	253 (30.5)
									
*High-risk infections*									
16	24	26	50 (6.7)	7^b^	4^b^	11 (13.9)	31	30	61 (7.3)
18	13	11	24 (3.2)	1	2^b^	3 (3.8)	14	13	27 (3.3)
31	5	5	10 (1.3)	2	3^b^	5 (6.3)	7	8	15 (1.8)
33	11	11	22 (2.9)	4^b^	5^a^	9 (11.4)	15	16	31 (3.7)
35	9	15	24 (3.2)	2	2	4 (5.1)	11	17	28 (3.4)
39	4	3	7 (0.9)	0	0	0	4	3	7 (0.8)
45	11	24	35 (4.7)	4^a^	1	5 (6.3)	15	25	40 (4.8)
51	5	9	14 (1.9)	0	2^b^	2 (2.5)	5	11	16 (1.9)
52	7	23	30 (4.0)	1	6^b^	7 (8.9)	8	29	37 (4.5)
56	8	8	16 (2.1)	0	2	2 (2.5)	8	10	18 (2.2)
58	8	16	24 (3.2)	1^b^	3^b^	4 (5.1)	9	19	28 (3.4)
59	3	3	6 (0.8)	0	0	0	3	3	6 (0.7)
68	4	3	7 (0.9)	0	1	1 (1.3)	4	4	8 (1.0)
73	3	5	8 (1.1)	0	4	4 (5.1)	3	9	12 (1.4)
82	3	3	6 (0.8)	1	1	2 (2.5)	4	4	8 (1.0)
Subtotal	118	165	283 —	23	36	59 —	141	201	342 —
									
*Low-risk infections*									
6	2	7	9 (1.2)	0	2	2 (2.5)	2	9	11 (1.3)
11	2	1	3 (0.4)	0	0	0	2	1	3 (0.4)
26	2	2	4 (0.5)	0	0	0	2	2	4 (0.5)
30	4	4	8 (1.1)	1	0	1 (1.3)	5	4	9 (1.1)
34	0	1	1 (0.1)	0	0	0	0	1	1 (0.1)
40	0	3	3 (0.4)	0	1	1 (1.3)	0	4	4 (0.5)
42	11	9	20 (2.7)	0	4^b^	4 (5.1)	11	13	24 (2.9)
43	2	10	12 (1.6)	0	1	1 (1.3)	2	11	13 (1.6)
44	0	0	0	0	1^b^	1 (1.3)	0	1	1 (0.1)
53	1	5	6 (0.8)	1	1	2 (2.5)	2	6	8 (1.0)
54	0	4	4 (0.5)	0	1	1 (1.3)	0	5	5 (0.6)
55	0	5	5 (0.7)	0	2	2 (2.5)	0	7	7 (0.8)
61	0	1	1 (0.1)	0	0	0	0	1	1 (0.1)
64	1	0	1 (0.1)	0	0	0	1	0	1 (0.1)
66	7	19	26 (3.5)	3^b^	5^b^	8 (10.1)	10	24	34 (4.1)
67	5	12	17 (2.3)	0	3	3 (3.8)	5	15	20 (2.4)
69	6	9	15 (2.0)	0	1	1 (1.3)	6	10	16 (1.9)
70	2	5	7 (0.9)	1	2	3 (3.8)	3	7	10 (1.2)
72	8	10	18 (2.4)	0	0	0	8	10	18 (2.2)
81	15	8	23 (3.1)	0	0	0	15	8	23 (2.8)
CP6108	3	3	6 (0.8)	0	0	0	3	3	6 (0.7)
Low-risk cocktail^c^	22	32	54 (7.2)	2	11^a^	13 (16.5)	24	43	67 (8.1)
Subtotal	93	150	243 —	8	35	43 —	101	185	286 —
X	30	0	30 (4.0)	3^b^	0	3 (3.8)	33	0	33 (4.0)
Total infections	241	315	556 —	34	71	105 —	275	386	661 —

a Includes two HSIL.

b Includes one HSIL.

c Includes HPV32,83,84,85,86,JC9710.

HPV=human papillomavirus; HSIL=high-grade squamous intraepithelial lesions; *N=*number; *X*=uncharacterised type.

**Table 2:** Cytological abnormalities and detection of high-risk HPV types by presence of suspected abnormalities at VIA/VILI among 822 women^a^ (Conakry, Guinea, 2006–2008)

	**VIA/VILI**					
	**Normal**			**Abnormal**		
**Cytology**	**High-risk HPV−**	**High-risk HPV+**	**All**	**High-risk HPV−**	**High-risk HPV+**	**All**
Normal	513	211	724	13	6	19
ASCUS	24	24	48	0	0	0
LSIL	4	11	15	0	1	1
HSIL	3^b^	11	14	1^c^	0	1
All	544	257	801	14	7	21

a Nine women without VIA/VILI results were excluded.

b Including one positive for HPV66.

c Positive for HPVX.

ASCUS=atypical squamous cells of undetermined significance; HPV=human papillomavirus; HSIL=high-grade squamous intraepithelial lesions; LSIL=low-grade squamous intraepithelial lesions; VIA/VILI=visual inspection with acetic acid or Lugol's iodine.

**Table 3:** ORs for HPV positivity and corresponding 95% CIs according to selected characteristics among 831 women (Conakry, Guinea, 2006–2008)

		**HPV-positive**					
**Characteristics^a^**	***N* women**	***N***	**(%)**	**OR^b^**	**95% CI**	**OR^c^**	**95% CI**
*Age group (years)*							
<25	214	120	(56.1)	1		1	
25–34	212	100	(47.2)	0.70	(0.48–1.02)	0.78	(0.51–1.21)
35–44	212	96	(45.3)	0.65	(0.44–0.95)	0.70	(0.44–1.12)
45–54	123	70	(56.9)	1.03	(0.66–1.62)	1.19	(0.71–2.00)
55–64	70	36	(51.4)	0.83	(0.48–1.42)	1.00	(0.54–1.84)
*χ*^*2*^_*1*_ *for trend*				*0.13;*	*P=0.716*	*0.51*	*P=0.473*
							
*Education*							
None	411	209	(50.9)	1		1	
Primary	176	86	(48.9)	0.93	(0.65–1.34)	0.91	(0.63–1.32)
Secondary or higher	244	127	(52.1)	1.03	(0.74–1.43)	0.94	(0.66–1.32)
							
*Marital status*							
Married	586	285	(48.6)	1		1	
Single	157	95	(60.5)	1.60	(1.04–2.45)	1.55	(1.00–2.41)
Widowed/divorced^d^	88	42	(47.7)	0.89	(0.55–1.43)	0.88	(0.54–1.42)
							
*Number of births*							
0	170	85	(50.0)	1		1	
1–2	226	122	(54.0)	1.32	(0.87–2.00)	1.45	(0.93–2.27)
3–5	225	103	(45.8)	1.06	(0.66–1.69)	1.32	(0.79–2.19)
⩾6	208	111	(53.4)	1.31	(0.77–2.26)	1.64	(0.93–2.90)
*χ*^*2*^_*1*_ *for trend*				*0.42;*	*P=0.518*	*2.11*	*P=0.146*
							
*Age at 1st intercourse (years)*							
⩾18	216	112	(51.9)	1		1	
16–17	455	231	(50.8)	0.92	(0.66–1.27)	0.90	(0.64–1.25)
≤ 15	133	64	(48.1)	0.78	(0.50–1.21)	0.78	(0.50–1.22)
*χ*^*2*^_*1*_ *for trend*				*1.12;*	*P=0.290*	*1.29*	*P=0.256*
							
*Lifetime sexual partners*							
1	454	227	(50.0)	1		1	
2	273	136	(49.8)	1.08	(0.78–1.48)	1.06	(0.77–1.46)
>3	76	46	(60.5)	1.71	(1.03–2.84)	1.64	(0.98–2.72)
*χ*^*2*^_*1*_ *for trend*				*3.23;*	*P=0.072*	*3.07*	*P=0.080*
							
*Polygamous husband*							
No	196	93	(47.5)	1		1	
Yes	621	323	(52.0)	1.31	(0.93–1.84)	1.30	(0.91–1.86)
							
*Husband's extra-marital sexual relationships*							
No	103	52	(50.5)	1		1	
Yes	701	357	(50.9)	1.11	(0.72–1.72)	1.10	(0.70–1.72)
							
*Use of hormonal contraceptive*							
Never	740	371	(50.1)	1		1	
Ever	90	50	(55.6)	1.30	(0.84–2.04)	1.15	(0.72–1.83)

a Some figures do not add up to the total because of a few missing values.

b Adjusted for age group, as appropriate.

c Adjusted for age group, ever/never married and lifetime number of sexual partners (1,2,>2), as appropriate.

d Including nine divorced women.

CI=confidence intervals; HPV=human papillomavirus; *N=*number; OR=odds ratios.

**Table 4:** Prevalence of selected HPV types in 70 HPV-positive women with ICC^a^ and 360 HPV-positive women with normal cytology (Conakry, Guinea, 2006–2008)

	**ICC (*N*=70)**	**Normal cytology (*N*=360)**	**ICC:normal cytology**
**HPV type**	**Total (%)**	**Total (%)**	**Prevalence ratio (95%CI)**
16	34 (48.6)	50 (13.9)	3.5 (2.2–5.5)
18	10 (14.3)	24 (6.7)	2.1 (0.9–4.6)
16/18	44 (62.9)	73 (20.3)	3.1 (2.1–4.6)
			
*Other high-risk types*			
33	2 (2.9)	22 (6.1)	0.5 (0.05–1.9)
35	3 (4.3)	24 (6.7)	0.6 (0.1–2.1)
39	2 (2.9)	7 (1.9)	1.5 (0.1–7.7)
45	13 (18.6)	35 (9.7)	1.9 (0.9–3.7)
			
Any high-risk type other than 16/18	25 (35.7)	169 (46.9)	0.8 (0.5–1.2)
Low-risk type	2^b^ (2.9)	219 (60.8)	0.05 (0.006–0.2)
X	2 (2.9)	30 (8.3)	0.3 (0.04–1.4)
Multiple infections	3^c^ (4.3)	119 (33.1)	0.1 (0.03–0.4)

a Seven HPV-negative ICC are not included.

b Includes one single HPV30 infection.

c Two HPV16+45 and one HPV16+69.

CI=confidence interval; HPV=human papillomavirus; ICC=invasive cervical carcinoma.
